# Applying the Multi-Scale Integrated Analysis of Societal and Ecosystem Metabolism (MuSIASEM) to characterize the society–agriculture–forest system: the case of Huayopata, Cuzco (Peru)

**DOI:** 10.1007/s10668-022-02457-6

**Published:** 2022-06-11

**Authors:** Juan José Cadillo-Benalcazar, José Carlos Silva-Macher, Norma Salinas

**Affiliations:** 1 Institute for Nature, Earth and Energy (INTE), Pontificia Universidad Católica del Perú (PUCP), Av. Universitaria 1801, 15088 Lima, Peru; 2 Departament of Economics, Pontificia Universidad Católica del Perú (PUCP), Av. Universitaria 1801, 15088 Lima, Peru

**Keywords:** MuSIASEM, Primary forest, Secondary forest, Agriculture, Cuzco

## Abstract

The divergence between the disappearance of primary forests and the appearance of secondary forests indicates a set of circumstances that simultaneously converge in what we called the society–agriculture–forest complex. Such circumstances vary between places and over time and are associated with internal factors—factors originating within the reference system—and external factors—factors originating outside the borders of the reference system—restrict the use of standard strategies for any reality. We present a quantitative model that helps to understand the relationships of the society–agriculture–forest complex as a whole. This comprehensive understanding will allow a clearer discussion of the positive and negative consequences of prioritizing actions on any of the system components. Our model establishes a set of quantitative relationships among: (i) the requirements of food and timber products for society to maintain its structure and functionality, (ii) the level of openness of the society with respect to other societies for the exchange of resources, and (iii) the interface between the economic productive systems and the ecological productive systems. To test the model, the case of Huayopata (Peru) was studied. Findings suggest that the abandonment of agricultural production and, particularly, of tea favors the appearance of secondary forests. However, projects by the Congress of Peru to reactivate the production of tea without adequate technological support to improve the current processes that use firewood for boilers would put the forestry system at risk. In addition, a potential worker 'pull factor' could reconfigure the food system and impact on the local agricultural sector.

**Supplementary Information:**

The online version contains supplementary material available at 10.1007/s10668-022-02457-6.

## Introduction

Year 2020 should have been a key year for the conservation of forests and biodiversity worldwide, in light of the fact that the deadline for several supranational commitments had expired:

- Sustainable Development Goal 15.1: "to ensure the conservation, restoration and sustainable use of terrestrial and inland freshwater ecosystems and their services, in particular forests, wetlands, mountains and drylands …" (United Nations, 2018 p. 67),
- Aichi Biodiversity Target 5: "the rate of loss of all natural habitats, including forests, is at least halved and, where feasible, brought close to zero, and degradation and fragmentation is significantly reduced” (CBD, 2010); and
- Goal 1 of the New York Declaration on Forests: "At least halve the rate of loss of natural forests globally by 2020 and strive to end natural forest loss by 2030" (UN Climate Summit, 2014).

Despite efforts made and a significant reduction in the net loss of forests—40% between the average of 1990–2000 and 2010–2020 decades (FAO & UNEP, 2020)—the objectives were not achieved (Secretariat of the Convention on Biological Diversity, 2020). For its part, the rate of deforestation is still so significant that it continues to jeopardize the conservation of biodiversity (Gibson et al., 2011) and the carbon sink capacity of forests (Harris et al., 2021), thus increasing the risk of impacting ecosystems to a point of no return (Lenton et al., 2019).

The expansion of the forest area, which partly compensates for deforestation, has seen an increasing trend in the appearance of so-called secondary forests. Some estimates suggest that these types of forests already occupy two-thirds of the global forest area and up to 70% of tropical forest areas (FAO, 2010 cited by Pain et al., 2020). Although there is no consensus on the definition of secondary forests (Chokkalingam & De Jong, 2001), their characteristics—capturing a greater amount of carbon compared to primary forests, reducing soil erosion, maintaining biodiversity, improving regulation of water flows, among others—make them important instruments for recovering forests' services lost through deforestation and reducing pressure on primary forests.

This divergence between the disappearance of primary forests and the appearance of secondary forests reflects a simultaneous confluence of a number of circumstances in what we called the society–agriculture–forest complex. Such circumstances, which vary over time and depending on the location, can be originated by:

- Internal factors—i.e., factors originating within the reference system. For example, people from a rural community (within society) who migrate to the city, reducing agricultural activity and pressure on forests.
- External factors—i.e., factors originating outside the borders of the reference system. For example, the demand for carbon obtained from *algarrobo* (carob) (*Prosopis pallida*) for the preparation of grilled chicken, intended mainly for the city of Lima, is negatively affecting the dry forests of northern Peru (OSINFOR, 2018).

This multiplicity of factors reflects the complexity that affects the society–agriculture–forest complex and restricts the use of standard policies and strategies for any reality. Since the beginning of the 1990s decade, payments for ecosystem services (PES) have become the most widely disseminated conservation strategy. According to Prokofieva, 2016, multilateral organizations such as the World Bank, the Global Environment Fund (GEF), the International Union for Conservation of Nature (IUCN), and the World Wide Fund for Nature (WWF) have become their most prominent promoters. However, the limited or adverse results of its implementation in various parts of the world have questioned its effectiveness. The study of Chan et al. (2017) has identified the following kinds of problems: (i) new negative externalities, (ii) loss of rights and confusion over responsibilities, (iii) displacement of intrinsic motivations, (iv) difficulties in the distribution of compensation, (v) high monitoring costs, (vi) limited applicability, and (vii) lack of understanding between the institutions and social groups involved.

These kinds of problems arise due to the implementation of policies and strategies that replace the uncertainty of such complex systems (society–agriculture–forests) with the certainty of human control procedures (Gunderson and Holling, 2002), thereby ignoring the socioecological context in which societies perform. Moreover, these public policies are not legitimate when the framing for the decision-making process reproduces the justification narratives of a particular social group, leaving out other perspectives (Skutsch & Turnhout, 2020). In this way, the results are not only ineffective, but are also the source of a number of social conflicts.

Then, framing the problems and causes of deforestation necessarily leads to the recognition of the contested perspectives and motivations of the various social stakeholders involved. Although these may be legitimate, they would need to be cross-checked through a properly informed dialogue—namely, by considering the complexity of the society–agriculture–forest system. Therefore, the design of public policies to face deforestation merits a participatory and inclusive debate, which gives quality assurance to the process and facilitates the adoption and appropriation of such policies. Thus, the perception of winners and losers among social stakeholders that favors the emergence of conflicts is avoided. In addition, participatory processes favor social stakeholders' awareness and preparation in the face of possible tensions between the socioeconomic system and the ecological system. For example, future scenarios suggest greater competition for land for food production, thereby endangering ecosystems (Tilman et al., 2011).

Promoting this properly informed dialogue requires an analytical tool capable of improving the understanding of the behavior of the society–agriculture–forest complex as a whole, without disregarding the fact that each component that constitutes the whole is, in turn, a system (Simon, 1962). This leads to describe the system and its relevant subsystems in their own structural elements that contribute to its functionality. This comprehensive understanding allows a clearer discussion of the positive and negative consequences of prioritizing actions on any of the system components. Addressing this type of questions: How does the implementation of policies aimed at promoting agriculture affect forests? What environmental and economic benefits would come from prioritizing forest protection? What effects do demographic changes have on agriculture and forests? Such an approach is aimed at confronting the social stakeholders involved with the uncomfortable knowledge of sustainability, a perspective that moves away from traditional approaches that do not pay due attention to social and environmental limits.

For this purpose, David Pimentel's analytical approach proves to be useful because it simultaneously represents the interactions among society, food, and energy. Exposing the uncomfortable knowledge of the expansion of human activity in the face of biophysical limits—incompatibilities between current food demand and conservation of ecosystems (David Pimentel & Pimentel, 2008)—and inconsistent policies and inconsistent strategies (for example, policies promoting biofuels to protect the environment, which have ultimately fostered deforestation and the loss of biodiversity Pimentel, 2012; Pimentel & Patzek, 2006)). The present study integrates the principles of Pimentel's work and the approach of the Multi-Scale Integrated Analysis of Societal and Ecosystem Metabolism (MuSIASEM) (Giampietro & Mayumi, 2000a, 2000b; Giampietro et al., 2012) quantitatively characterizing the society–agriculture–forests complex. The MuSIASEM can represent different dimensions (social, economic, and environmental), considering multiple scales and levels of analysis. This approach has been applied in different sectors, such as mining (Silva-Macher, 2015), energy (Di Felice et al., 2019; González-López & Giampietro, 2018; Velasco-Fernández et al., 2015, 2018), and agriculture (Cadillo-Benalcazar, Giampietro, et al., 2020; Cadillo-Benalcazar, Renner, et al., 2020; Renner et al., 2020). In the forestry, the MuSIASEM has been used to create negentropic indicators to characterize the degree of disturbance of different biomes (Lomas & Giampietro, 2017). In this paper, the MuSIASEM will be applied for the first time to analyze the society–agriculture–forests complex, with an emphasis on secondary forests.

To illustrate the relevance of this approach, this paper discusses the case study of Huayopata in Cuzco (Peru), which shows how public policies for tea production interact with the complexity of the society–agriculture–forest system. In this regard, the MuSIASEM approach is applied in order to understand the state of the system in its pre-COVID-19 stage.

The remainder has the following structure: section two describes the historical context of Huayopata, section three presents the methodology, section four summarizes the main results, section five focuses on the discussion, and section six sets out conclusions.

## Historical context of Huayopata

The district of Huayopata was created on January 2, 1857, and belongs to the La Convencion province, which is located in the department of Cuzco, in southern Peru. Huayopata has an area of 531 square kilometers and is one of the country's leading tea producers. According to RUNAQ, 2017, Benjamin de la Torre led the introduction of tea as a crop in the first decade of the twentieth century, but upon his assassination, the plantations were abandoned. In 1927, Augusto B. Leguía's administration promoted the recovery of the activity by hiring Sri Lankan experts to train farmers, which gave rise to large tea plantations. In the 1940s, the Huyro Tea factory and later the Amaybamba factory were created (ibid). Summing up the description of Huizer, 1983: labor shortages meant that landowners would encourage immigrants to come in return for providing them with land to work on their own crops. Those first migrants were referred to as *arrendires*. However, the economic prosperity achieved by *arrendires* through coffee caused them to neglect the tea plantations. This motivated landowners to exert pressure on *arrendires* to work at the tea plantations. Under these circumstances, *arrendires* called in new immigrants and provided them with a fraction of their land in order to help them with the work assigned by landowners and avoid their neglecting the coffee growing. These new migrants were called *allegados* (Huizer, 1983). The exploitative working conditions fostered by landowners in the region resulted in the rebellion of the peasants, who joined forces to confront such injustices. There had reportedly been several instances of violence. This led Ricardo Pérez Godoy's administration to institute the agrarian reform process in 1963–1964, transferring ownership of inefficiently exploited lands to the peasants. Although this process included the Huayopata land, it did not significantly affect the areas intended for the cultivation of tea (Huizer, 1983). Moreover, the 1969 Agrarian Reform Decree, enacted by Juan Velasco Alvarado's dictatorial government, allocated the land to peasants under the slogan 'the land is for those who work it'. This had a number of negative consequences. On the one hand, it resulted in an unequal distribution of the land, in such a way that *arrendires* and some leaders of the peasant associations received a larger extension of land compared to that received by *allegados*, which created conflicts among them (Guerra García, 1987). On the other hand, the agrarian reform increased deforestation—while landowners maintained the forest resource, the new owners began to expand their cultivation areas (Farfán & Hurtado, 1996).

During the 1960–1990 period, tea production remained around 1150 tons per year (RUNAQ, 2017); however, the need for energy to keep the tea factory cauldrons operational was also a driver of deforestation (Farfán & Hurtado, 1996). Another consequence of the agrarian reform was the creation of cooperatives to help members in the production and marketing of their product. Therefore, the Huyro and Amaybamba tea factories were transferred to the Central de Cooperativas Agrarias Te-Huyro in 1970 (RUNAQ, 2017). Nevertheless, lack of management skills, government corruption and the start of the internal armed conflict adversely impacted on tea production, drastically reducing the economic income for farmers. According to Huizer, 1983, in the 1990s, Alberto Fujimori's dictatorial rule liberalized the domestic market by allowing the entry of tea from Argentina, which dragged down the already fragile national tea economy. In recent years, the government has again been promoting domestic tea production through the enactment of new bills (3821/2018-CR and 5936/2020-CR), which facilitate the acquisition of equipment and infrastructure to obtain organic production certificates. In this way, it promotes tea exports to the US market.

## Materials and methods

### Theoretical foundations

Professor David Pimentel was at the forefront in describing the relationship between consumption patterns and environmental pressures. In our opinion, Pimentel made pioneering efforts to carry out the so-called Nexus analysis—which gained relevance since the 2011 Bonn Conference (WEF nexus, 2011)—as he began to relate food production to energy (Pimentel et al., 1973) and, later on, to water (David Pimentel & Pimentel, 2008). Giampietro's and Mayumi's MuSIASEM improves Pimentel's approach via the operationalization of a quantitative representation of such relationships (MuSIASEM grammar) (Giampietro & Mayumi, 2000a, 2000b). In this sense, the MuSIASEM approach adds: Firstly, the Hierarchy Theory (Ahl & Allen, 1996) to describe the Socio-Ecological System (SES) and its constituent components across a hierarchical organization. Secondly, the Flow/Fund Theory (Georgescu-Roegen, 1971) to represent what the *system is* and what the *system does*. Thirdly, the concept of Societal Metabolism to establish the metabolic pattern, that is, the profile of energy and matter exchange that occurs between the components of the SES, in order to maintain and reproduce its structure and functions (Giampietro et al., 2012). Finally, it incorporates the Relational Analysis (Rosen, 1991) to evaluate the intrinsic and extrinsic linkages of the system, the subsystems and their environment.

### Construction of the MuSIASEM grammar

**Fig. 1:**
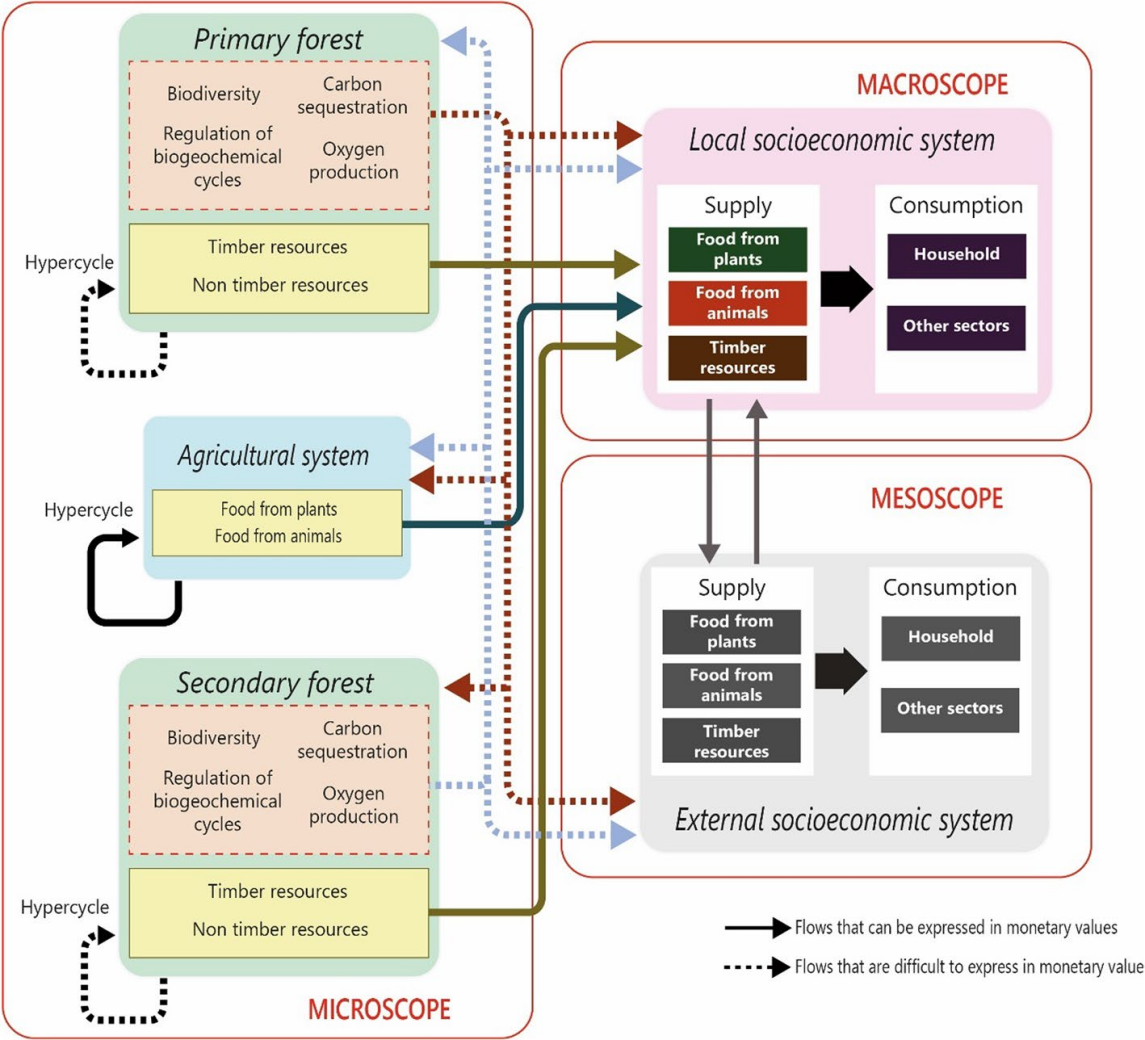
Grammar to characterize the society–agriculture–forest complex

The construction of the MuSIASEM grammar is part of the pre-analytical phase, which is constantly evaluated in an iterative and reflexive process. The grammar entails defining a set of semantic categories and establishing the relationships that occur among them. Figure 1 illustrates the grammar proposed for the present study, which describes the SES including five components: primary forest, secondary forest, agricultural sector, local socioeconomic system, and external socioeconomic system. In addition, the grammar shows the interactions of inputs and outputs among these components, which are described using different value systems. For instance, some of the exchanges can be valued in monetary terms—such is the case of wood extracted for cash income—and other exchanges can be valued in biophysical terms—for example, firewood used for cooking purposes. Moreover, the grammar includes the relationship called *hypercycle*. This notion is adopted from the work of (Ulanowicz, 1986) and (Eigen & Schuster, 1979), which refers to the processes by which flows of energy and matter are synthesized. That is, the supplying party. Subsequently, these flows are used by the components of the system itself and/or by the systems with which they interact (such consumers belong to the dissipative part). In Fig. 1, the hypercycle refers to the flows of matter and energy that are produced by the same system and are required for its own maintenance, thereby providing the system (forestry and agriculture) the duality of being a productive and dissipative system. For example, the eggs used for chicken reproduction, or the leaf litter from trees used to provide organic matter to soils.

This relational analysis, expressed in the grammar, facilitates the study of the double causality that is a characteristic of complex systems:

- *The downward causality* that describes the influence exerted by the final consumption component on the production system and this, in turn, on the environment (Campbell, 1974). For example, an increase in beef demand will imply greater livestock production and therefore major environmental pressure (consumption → supply → ecosystem).
- *The upward causality* that describes the biophysical limitations imposed by the environment that will influence final consumers (Grene, 1987). For example, the total forest loss will lead to a shortage of firewood, which will impact final consumers who will look for alternative energy sources (ecosystem → supply → consumption).

The relevance that this relational analysis bears to sustainability is that it verifies what you want to do against what you can do. For a better understanding, the MuSIASEM approach proposes the use of three lenses:

- *The Macroscope* lens analyzes the final consumption of resources by the household sector and other economic sectors, such as local tea production.
- *The Mesoscope* lens studies the exchange of resources between the local socioeconomic system and external socioeconomic systems, which represents the system's degree of openness.
- *The Microscope* lens analyzes the interface among economic productive systems and ecological productive systems. For this purpose, the MuSIASEM approach introduces the concept of *processor* that identifies the metabolic pattern (see Cadillo-Benalcazar, Giampietro, et al., 2020; Cadillo-Benalcazar, Renner, et al., 2020; González-López & Giampietro, 2018 for further details.)

Giampietro et al., (2012) state that in order to carry out an adequate grammar of the SES, a definition of the system and its behavior must first be offered, that is, providing answers to a couple of questions: what is the system? And, what does the system do? According to these authors, the system represents that which is to be preserved, while its behavior is related to the metabolic profile that the system requires for its maintenance and reproduction. To express this analytically, the MuSIASEM adopts the language of *funds* and *flows* proposed by Georgescu-Roegen, 1971. *Fund* elements are those that do not change their attributes during the analytical representation, therefore, those identify what you want to preserve (the system). For example, human activity, Ricardian land, and power capacity. On the contrary, *flow* elements change their attributes during the analytical representation; consequently, they identify the inputs and outputs that the system requires to maintain their structure and functions. For example, water, food, energy, and waste, among others.

Table 1 shows the elements of *flows* and *funds* used to characterize the system. From these elements, the characterization of each of these components is made through extensive variables that will provide information on the size of the system (*fund* elements) and intensive variables that provide information on the metabolic profile of the system (*flow/fund or flow/flow* ratios).

**Table 1:** The elements of flows and funds used to characterize the system

System	Component	Element	Category	Unit
Forest	Secondary forest	Biomass	Fund	kilogram, ton
		Timber resources	Flow	kilogram, cubic meters
		Land	Fund	hectare
	Primary forest	Biomass	Fund	kilogram, ton
		Timber resources	Flow	kilogram, cubic meters
		Land	Fund	hectare
Agriculture	Agriculture sector	Plant-based products	Flow	kilogram, ton
		Animal-based products	Flow	kilogram, ton
		Animal herd	Fund	No. of animals
		Blue water	Flow	cubic meters
		Green water	Flow	cubic meters
		Land	Fund	hectare
Society	Productive sector	Human activity	Fund	hours
		Power capacity	Fund	kilogram, ton
	Household sector	Human activity	Fund	No. of people
		Plant-based products consumed	Flow	kilogram, ton
		Animal-based products consumed	Flow	kilogram, ton
		Timber products consumed	Flow	kilogram, ton

### Data collection

To characterize the agricultural system, data on human activity, use of fertilizers and pesticides per crop were obtained from Agencia Agraria La Convención, 2020a. In some cases, such data were adjusted on the recommendation of experts. In the absence of specific data for blue and green water for agricultural production in Huayopata, data from Cuzco were used. Such data were obtained from Mekonnen & Hoekstra, 2010. Data on production, harvested and sown area, yield and prices of agricultural crops were obtained from the Agencia Agraria La Convención, 2020b. Data relating to hectares used are the average of years 2015, 2016 and 2017, except for data related to tea, which has been sourced from (local tea producer, personal communication).

In the absence of information on food consumption, the food pattern of Cuzco was used as a reference (INEI, 2012) and adjusted to the food pattern of Huayopata through local experts. Due to the lack of information, it was assumed that 80% of yucca, 20% of corn, 50% of onion, 50% of potato and 20% of tomato consumption derive from local production. In the case of coffee and tea, we assumed that local consumption data are not representative. To estimate the import and export of food, the adjusted food pattern of Huayopata was used and multiplied by the population and by 365 days. Thus, the annual feed requirement was established. Subsequently, processed foods were transformed into their primary products. Finally, the annual requirement of primary foods was contrasted against production.

If production was greater than the requirement, the surplus was assumed as an export. Alternatively, if the requirement was greater than production, the shortage was assumed as an import.

Forestry engineer Julio Lovera Fernández prepared the production costs of secondary forests (see Supplementary Material). Data relating to households that consume firewood for cooking purposes were obtained from INEI, 2018. The population and economically active population data were obtained from INEI, 1993, 2008, 2018. Data concerning biomass of secondary forests and primary forests were collected from Aragón et al., 2021. Tea production and firewood consumption data for the tea factory were gathered from (local tea producer, personal communication).

## Results

### General diagnosis

**Fig. 2:**
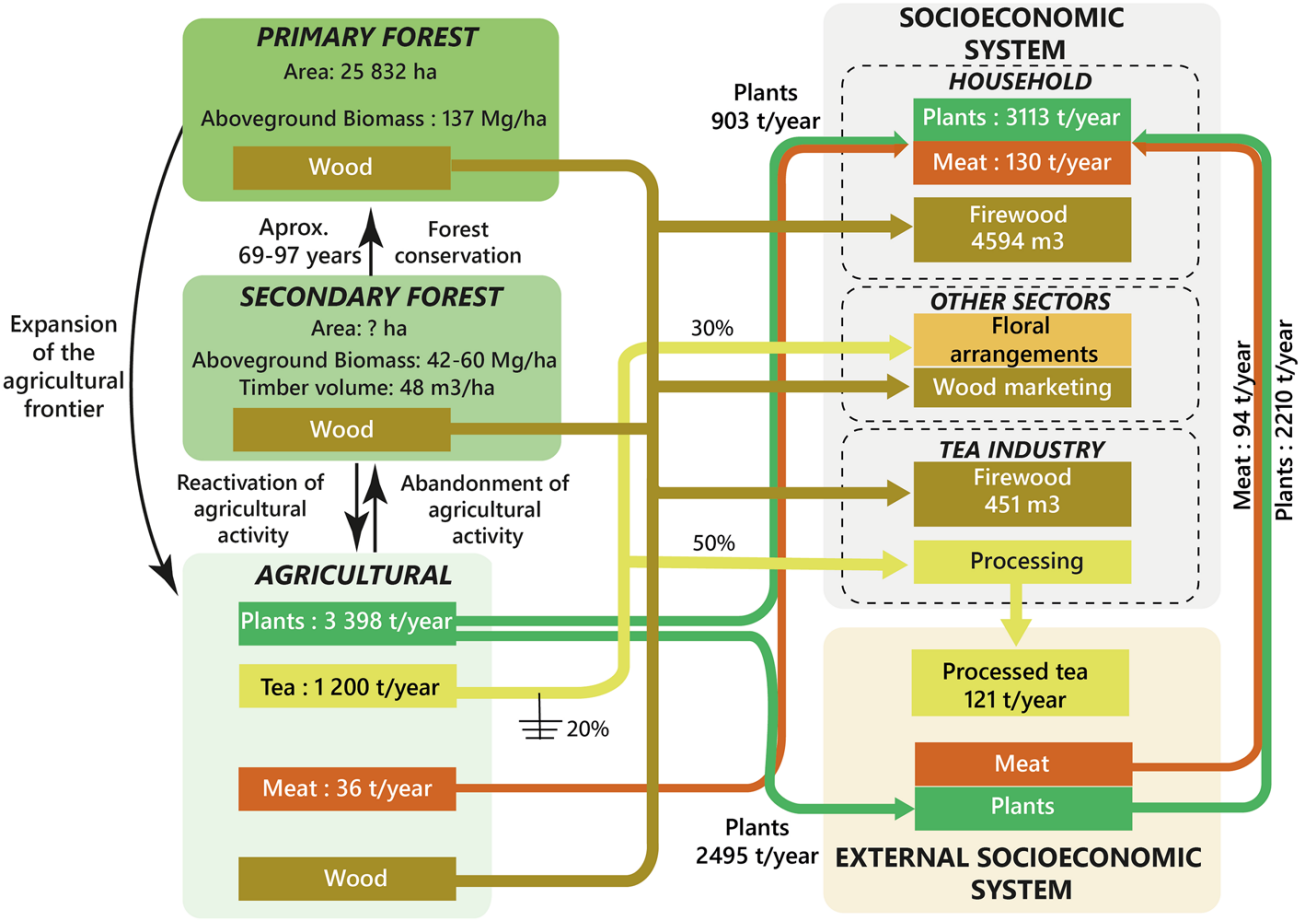
General diagnosis of Huayopata

The forest–agriculture–society complex includes five compartments: primary forest, secondary forest, agriculture system, local socioeconomic system, and external socioeconomic system (see Fig. 2). The behavior of each of them is positively or negatively impacted by the behavior of the others. However, it will be human activity (in this case Huayopata's) that exerts an important influence on these components. For instance, the cessation of agricultural activity may give rise to secondary forests. Moreover, if Huayopata opts for the protection of secondary forests, these would need almost a century to achieve some attributes of primary forests. On the other hand, the expansion of agricultural land can reduce the forest area in a short period of time. Probably, secondary forests will be the first to be addressed due to their proximity followed by primary forests. These transitions are represented by the black arrows in Fig. 2. The conversion of forest and agricultural areas into urban areas can also occur. However, the reverse process is less likely.

Huayopata's socioeconomic system requires resources and energy to maintain its structure and functionality. In this sense, the agricultural sector is the component that provides food for domestic consumption and for export to obtain economic benefits—noting export as the output of biophysical resources outside the limits of Huayopata. Therefore, it can be either national or international. Our estimates suggest that the agricultural sector supplies 910 tons/year of plant-based food (green arrows) and 36 tons/year of animal-based food (orange arrows) intended to ensure food security. However, such amounts are not sufficient in terms of quantity and food type. For this reason, Huayopata needs to import around 2200 tons/year of plant-based food (green arrows) and 104 tons/year of animal-based food (orange arrows) to cover internal consumption. In terms of food self-sufficiency, Huayopata is 29% self-sufficient in terms of plant-based food and 28% self-sufficient as regards animal-based food. The above percentages suggest a significant food dependency.

Despite such food dependency, some foods (mainly fruits) are exported—approximately 2495 tons/year. In view of the importance of tea in the area, this is represented individually. Figure 2 evidences that tea is mainly intended for export of processed tea (50%), flower arrangements (30%), and 20% is wasted. Such percentages are approximations obtained from (local tea producer, personal communication). From these data, it is estimated that export of processed tea stands at 121 tons/year. The importance of this overview is that any change in patterns of food consumption, improvement of food self-sufficiency, Huayopata's economic activities, or in commercial dynamics of food and tea will have implications on the agricultural system. Consequently, the behavior of the agricultural system will impact the conservation of forests.

Diagnosis through the macroscope, mesoscope, and microscope lenses is described in detail in the following sections.

**Fig. 3:**
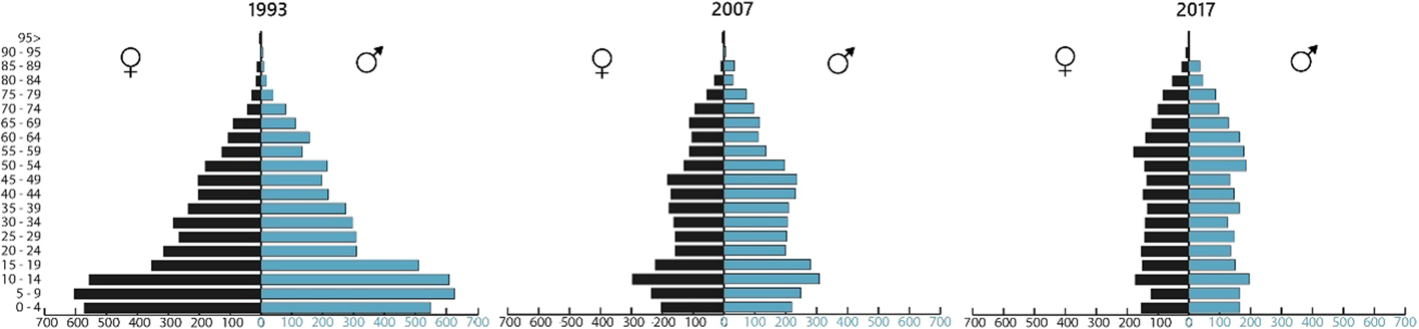
Demographic changes in Huayopata between 1993 and 2017 (INEI, 1993, 2008, 2018)

Demand for biophysical resources and productive activities is linked to population dynamics. For instance, an increase in population will require more resources, such as food and firewood for cooking purposes (provided the same food preparation technologies are maintained). In contrast, a reduction of the population will not only mean less demand for resources, but also a smaller availability of labor force. In this sense, it is important to characterize the demographic changes of recent decades, which determine the viability of local production. Huayopata's population has decreased by around 46% in the last three decades (see Fig. 3). Grounds for this demographic change being low income in agriculture, lack of employment alternatives, and internal armed conflict. This displacement of people has also had an impact on the workforce, which has decreased by 30%. In the agricultural and forestry sector, the number of workers went from 1545 in 1993 (INEI, 1993) to 1077 in 2017 (INEI, 2018). These activities continue to be the most predominant (employing 55% of the workers in Huayopata). However, the age group parameter has seen notable changes: the number of workers aged 15–29 has decreased by 68%, whereas the number of those aged 30–44 has decreased by 48%. For its part, the number of workers aged 45–64 has increased by 14%. One possible explanation lies in the limitations that the elderly population has to move to the cities and integrate into other economic activities. If these trends continue in the future, there will be a problem of workforce availability.

### Macroscope

Demand for food and firewood by the components that constitute the local socioeconomic system is analyzed through this lens.

#### Household sector

**Fig. 4:**
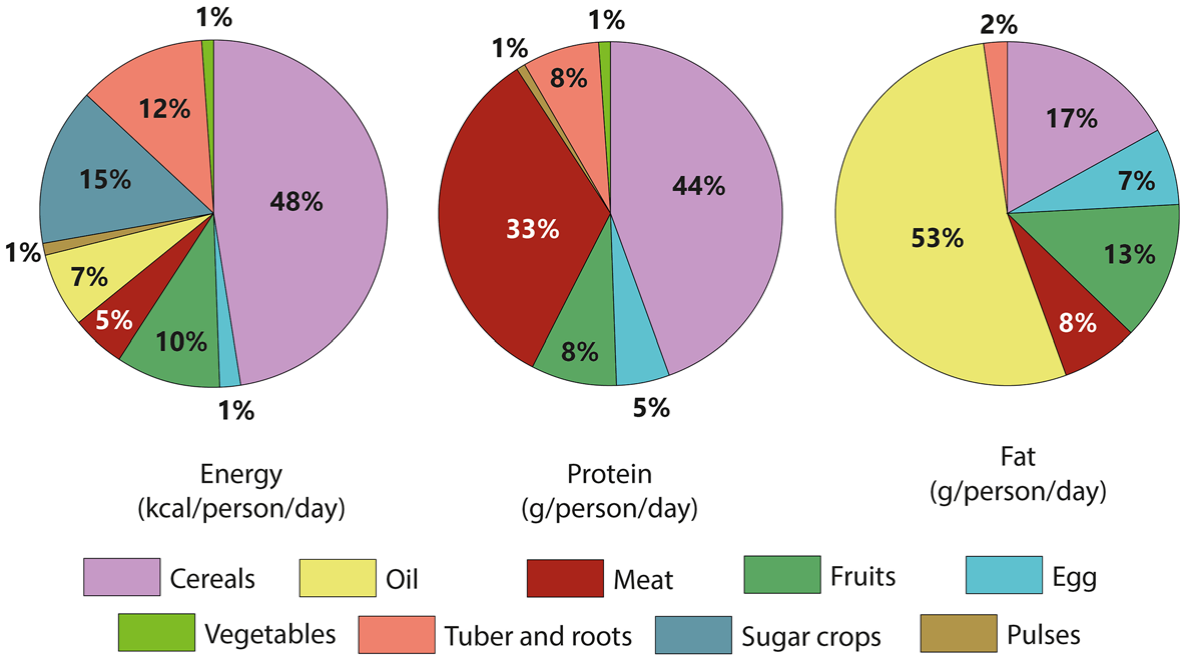
Energetic and macronutrient profile of Huayopata

An important aspect in the well-being of the population is adequate nutrition. Therefore, the integration of nutritional profile indicators is of the essence, especially, when 28.4% of the population live in poverty and 4.8% in extreme poverty (CEPLAN, 2017). Figure 4 shows Huayopata's nutritional profile: 1796 kcal of food energy per capita; 48 g of proteins per capita; and 28 g of fats per capita. As a reference, given that the values are not comparable due to methodological differences, the average reported for rural areas in Peru is as follows: 1804 kcal per capita for energy, 55 g per capita for proteins, and 36 g per capita for fats (INS, 2018). The key being that any policy seeking to improve people's nutrition—whether in terms of food quantity or quality—will have an impact on agricultural systems, ecosystem (forest system), and labor force (*downward causality).*

This accounting system has the advantage of adding/disaggregating items according to analytical purposes, for example:

- By the type of food groups, cereals account for 48% of total food energy and 44% of total proteins, while oils account for 53% of total fats. Meat products account for 33% of total proteins but have their contribution is lower in terms of energy and fats.
- By food: among the main products consumed in Huayopata are those derived from wheat (125 g/person/day) and rice (99 g/person/day), which are not locally produced. Therefore, the environmental pressure associated with wheat and rice is externalized. For their part, tubers and roots (212 g/person/day), legumes/ pulses (4 g/person/day) and vegetables (52 g/person/day) are produced mainly for self-consumption. In addition, fruits, which record a 381 g/person/day consumption, are produced mainly for cash income purposes via market trade.

In the household sector, there is an energy vulnerability problem. According to INEI, 2018, around 87% of the population uses firewood for cooking purposes (~ 4176 persons). When multiplied by the benchmark used by government institutions on fuelwood consumption—1.1 m^3^/person/year (La Torre-Cuadros and Mentón, 2016)—we obtain an annual 4594 m^3^/year demand for firewood. A crucial aspect that must be considered is the comparison of scales—while use of firewood for cooking purposes is daily, the renewal time of firewood is counted in periods of years that will vary according to the species. The components of the SES providing firewood are the agricultural sector and forest system (primary and secondary forest). However, there are no data to trace the origin of firewood. In some cases, due to efforts required to collect firewood, it is bought from merchants who extract it from other locations. Importing firewood—i.e., the influx of biophysical resources within the limits of the study area, be it from a national or international origin—from other locations only transfers the deforestation problem elsewhere (outsourcing). Merma & Julca, 2012 claim that natural forests are the reserve of firewood and wood for families in the Alto Urubamba, including the district of Huayopata.

#### Tea industry

The tea industry was in decline due to its low profitability until a recent 80% increase in the price of tealeaves—from 0.5 to 0.9 Soles/kg—as well as the expectations created by two congress bills for the promotion of national production of tea (local tea producer, personal communication). Currently, approximately 250 hectares are producing 1200 tons of fresh leaves per year, of which 50% are for tea production, 30% are for flower arrangements and the remaining 20% is wasted (ibid.). From the 600 tons per year that make it to the industry, 121 tons of black tea per year are obtained, which are sold in the Peruvian coastal markets. However, the profitability of that production depends on cheap energy to process it. Ortega Loaiza (2013) detected that at the Central de Cooperativas Agrarias Té Huyro ltda. 43, located in Huayopata the use of firewood from deforestation in surrounding areas influenced the company's financial statements. In the past, it has also been suggested that tea production was linked to deforestation (Farfán & Hurtado, 1996). Therefore, it was assumed that firewood used by this industry comes from the forest area. Consequently, it is estimated that in order to obtain 1 kg of black tea, approximately 2.70 kg of firewood needs to be burned (local tea producer, personal communication). This value is within the range of 1.67 to 8.2 kg of firewood per kilogram of black tea indicated by (Taulo & Sebitosi, 2016) for Malawi's tea industry. For current production level, demand for firewood is 327 tons—using a conversion factor of 725 kg/m^3^ (Malleux, 2000)—and the volume stands at 451 m^3^/year.

### Mesoscope

**Fig. 5:**
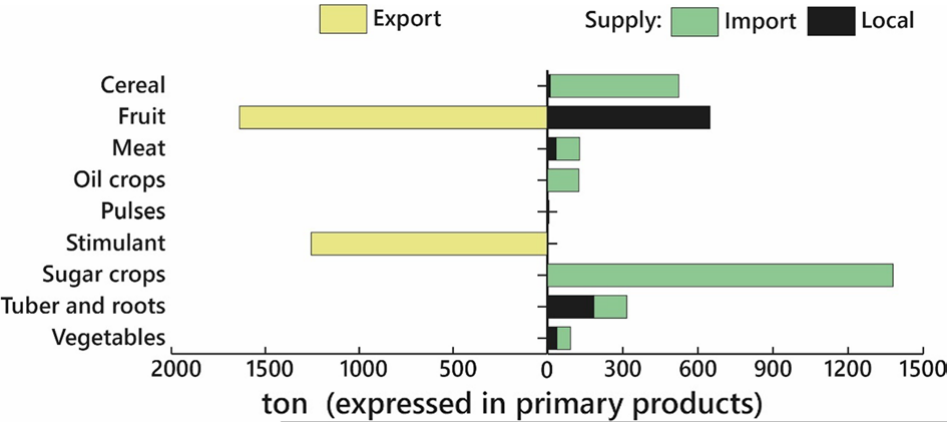
Level of openness of the Huayopata food system

As regards the exchange of food, Fig. 2 shows Huayopata's food dependency. However, through this lens it is possible to identify the degree of dependence according to the type of food or by food. Figure 5 evidences a complete dependence on sugar and oil products. It is clear that improving local production of such products is not possible, unless a processing plant is installed locally on site and the ecological conditions enable food used as raw material to be grown. The same is true for cereals, which are generally imported (around 98%). In contrast, the level of dependence on tubers and roots, and vegetables is lower. As for fruits, there is a surplus that is ultimately exported.

In respect of tea, current export levels stand at approximately 121 tons/year. However, tea improvement plans project further exploitation. In this context, a greater future demand for tea will have an impact on the expansion of tea crops and indirectly place greater pressure on the agriculture–forest system to obtain firewood for processing.

The importance of this type of information lies in the fact that it allows us to analyze the capacity for self-reliance and evaluate changes in behaviors. For example, the immigration 'pull factor'—the reactivation of tea production will result in a greater demand for food, which can be of local or external origin. Currently, the resumption of economic activities that had been halted due to the COVID-19, the increase in fuel prices, the increase in freight costs, the limitations on exports imposed by fertilizer-producing countries, and the conflict between Russia and Ukraine are driving up food and fertilizer prices. This situation jeopardizes food security in the district and its main productive activity, namely agriculture.

### Microscope

This lens is aimed at analyzing the interface of production processes against the socioecological system. The MuSIASEM facilitates the organization of information at different scales and levels to select extensive *fund* and *flow* variables (see Table 2) and build indicators based on the *flow/fund* and/or *fund/fund* relationship (see Table 3). The combination of both variables provides information on the size and performance of production systems.

**Table 2:** Extensive variables by agricultural product

Group	Food	Crop (t)	Land (ha)	Green water (m^3^)	Blue water (m^3^)	Human activity (h)	Fertilizer (kg)
Cereals	Maize	11	4	13,183	1592	1690	1402
Fruits	Avocado	216	24	133,005	54,861	19,200	5040
	Custard apple	66	9	50,413	41,282	7543	1980
	Lemon	41	4	20,891	13,133	2460	902
	Mandarin	65	7	21,966	10,635	1508	1040
	Mango	64	13	31,489	22,604	6400	0
	Orange	404	40	202,566	109,395	9373	6464
	Pineapple	184	23	48,933	8222	9200	5520
	Papaya	31	4	13,786	5685	2746	0
	Plantain	838	75	566,676	188,114	28,990	16,005
	Sweet granadilla	593	85	133,404	105,021	67,771	17,790
Pulses	Beans	7	5	5546	437	2800	607
Tubers	Cassava	50	4	30,899	0	3456	1190
	Potato	120	10	10,399	14,842	8240	3400
	Uncucha	16	2	7452	1119	1400	0
Vegetables	Tomato	6	negl	1006	0	192	67
	Onion	31	1	nd	nd	844	311
Stimulants	Coffee	656	937	8,319,425	0	442,331	226,789
	Tea	1200	250	3,292,230	1,820,093	38,000	0

nd = no data, negl=negligible

**Table 3:** Intensive variables by agricultural product

Group	Food	Crop/Land (t/ha)	Labor/Land (h/ha)	Fertilizer/Land (kg/ha)	Green water/Land (m^3^/ha)	Blue water/Land (m^3^/ha)
Cereals	Maize	2.50	400	332	3121	377
Fruits	Avocado	9.00	800	210	5542	2286
	Custard apple	7.00	800	210	5347	4378
	Lemon	10.00	600	220	5095	3203
	Mandarin	10.00	232	160	3379	1636
	Mango	5.00	500	0	2460	1766
	Orange	10.00	232	160	5014	2708
	Pineapple	8.00	400	240	2128	357
	Papaya	7.00	620	0	3113	1284
	Plantain	11.10	384	212	7506	2492
	Sweet granadilla	7.00	800	210	1575	1240
Pulses	Beans	1.50	600	130	1188	94
Tubers	Cassava	13.00	900	310	8046	0
	Potato	12.00	824	340	1040	1484
	Uncucha	8.00	700	0	3726	560
Vegetables	Tomato	30.00	1000	350	5241	0
	Onion	39.91	1080	398	nd	nd
Stimulants	Coffee	0.70	472	242	8877	0
	Tea	4.80	152	0	13,169	7280

nd, no data

**Fig. 6:**
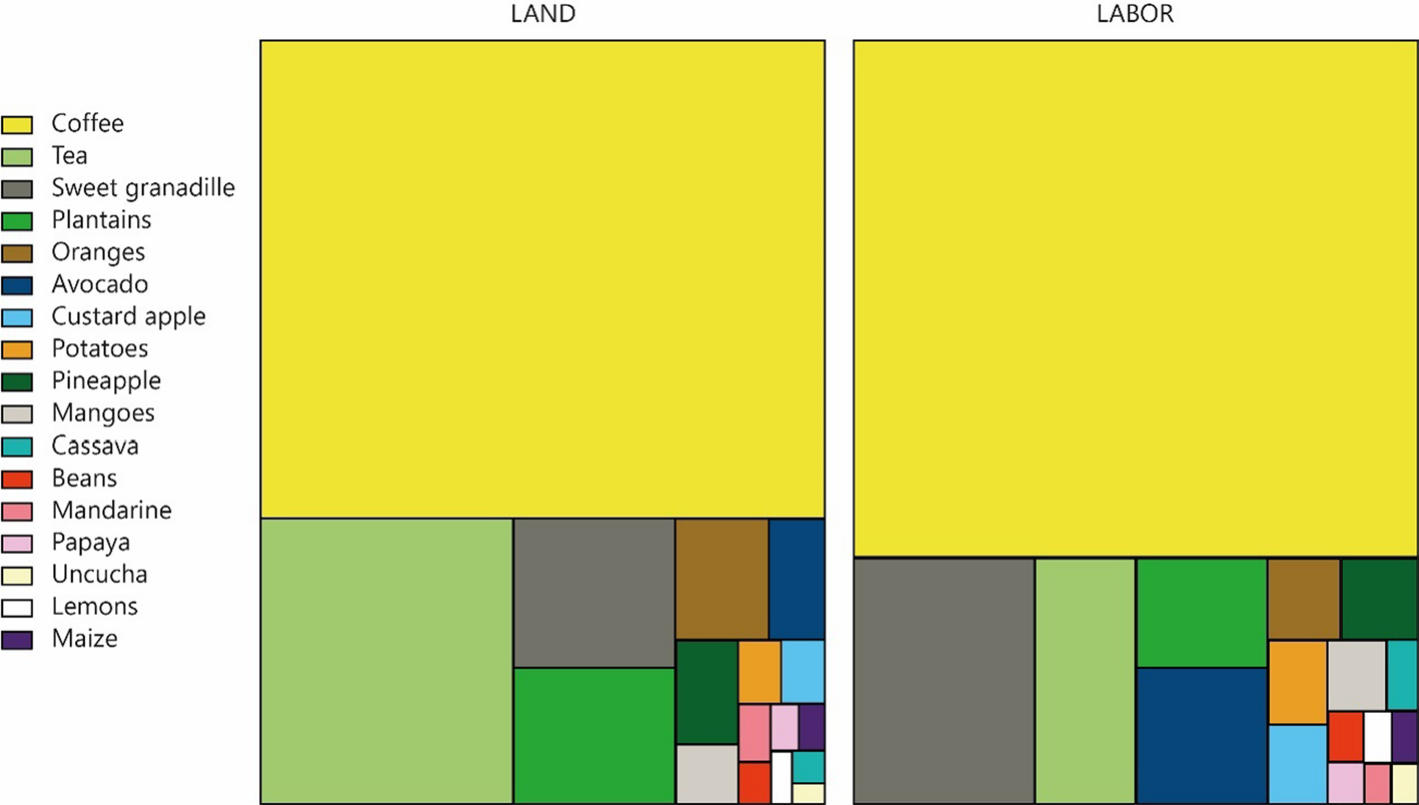
Breakdown of extensive land and labor variables

Table 2 shows the characterization through extensive variables of the agricultural system of Huayopata. This evidences that tea is the crop that records the highest production (1200 t), whereas coffee requires most labor (around 442,000 h) and green water, i.e., rainwater (around 8 million m^3^). The importance of green water—which is beyond human control—in agricultural production can also be observed. Conversely, fruit trees (especially plantain, orange, sweet granadilla and avocado) are most dependent on blue water. As for land use, it was noted that the greatest use is for products to be exported, that is, stimulants and fruits. In this sense, when food is exported, the biophysical resources used for its production are also exported. Figure 6 shows that a larger area used for a crop does not necessarily apply the same pattern for other inputs. In this case, two basic elements are compared: land and labor. It must also be noted that in Huayopata, which has a multi-crop system, it is not always possible to add the hectares by type of crop, because double accounting can be obtained.

This type of information also helps to plan food production, based on the amount of labor that is required. In previous sections, we noted that Huayopata loses its labor force due to migration. Thus, it is suggested that crops such as coffee and granadilla are the most impacted. The same is true for fertilizers, which are highly needed for coffee and fruits such as coffee sweet granadilla and plantain. Currently, the Cuzco region (where Huayopata is located) is experiencing a farmers' strike due to this input's price increase.

Table 3 shows indicators that help understand performance in the use of biophysical resources of each crop. For example, vegetables record a higher production per unit of land, whereas stimulants register a lower production per unit of land. Linking these results to those delivered in Table 2 suggests that increasing production under the same production system requires a greater amount of land compared to other crops. This is particularly the case with coffee, where the yield fails to reach one ton per hectare. Likewise, vegetables require a greater amount of fertilizers per unit of land. The low prices of tea have caused the current production system to be minimal, and this is why fertilizers are not applied. In addition, demand for labor has been covered with labor exchange. That is, farmers have helped each other in the agricultural tasks. These indicators also make it easier to project the consequences of some changes. For example, an improvement in fertilization will entail greater production and therefore greater demand for labor in the harvest phase.

**Fig. 7:**
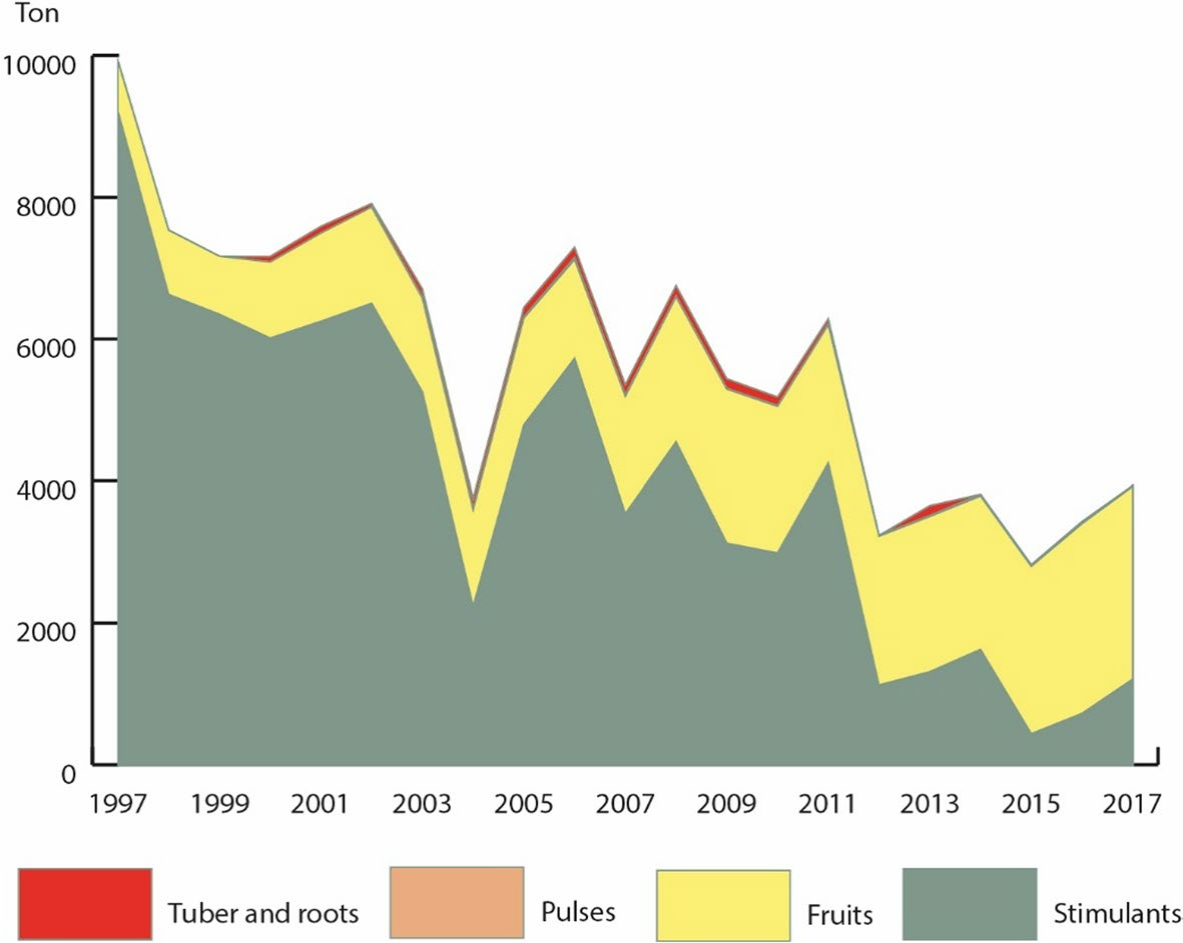
Evolution of agricultural production in Huayopata (1997–2017)

The low importance given by government institutions to secondary forests makes it difficult to obtain reliable data on their land area. Some indications suggest a growth of secondary forests. According to MINAM, 2020 records, secondary vegetation cover rose from 4370 ha in 2000 to 4529 ha in 2016. In addition, agricultural production has declined since 1997 (see Fig. 7), and yields (production/area) of the main products have either remained stable or decreased (Agencia Agraria La Convención, 2020b).

Secondary forests in Huayopata are distributed in a patchwork of agricultural areas—the reason for this being that they appear upon the abandonment of agricultural activities. However, this situation makes secondary forests vulnerable to the resumption of activities and the extraction of wood. For this reason, it is important for the social stakeholders involved to discuss the future of secondary forests. In this sense, the MuSIASEM in simulation mode serves as the basis for the construction of multi-criteria indicators that contribute to deliberation. For example, Table 4 shows possible quantitative (based on Flow and Fund elements) and qualitative (based on perceptions) indicators. In this table, two scenarios are considered: one focusing on the conservation of forests, and the other centering on the extraction of wood. In the first scenario, preserving secondary forests brings ecosystem benefits that are difficult to transform into monetary values. Such is the case of the recovery of biodiversity, regulating hydrological cycles, etc. For its part, the second scenario, i.e., extracting wood for trade purposes will have an economic potential of 4000 Soles/ha (1042 US$/ha), but it can take years or even decades to obtain new wood depending on the species and the desired wood volume—the plot of land used to estimate economic values is 30 years old (Aragón et al., 2021). Meanwhile, agricultural products have much shorter harvest cycles.

**Table 4:** Indicators for two possible scenarios

Scenario	Criteria	Value	Unit	Source
Secondary forest conservation	Aerial biomass	42–60	Mg/ha	Aragón et al. (2021)
	Time to reach maturity	100+	Year	Aragón et al. (2021)
	Biodiversity conservation	High		Working group evaluation
	Conservation of the hydrological cycle	High		Working group evaluation
Extraction for timber markets	Profit margin in the timber market	4000 (1042)	Soles/ha (US$/ha)^a^	Estimated by Eng. Julio Lovera Fernández
	Hours of work provided	540	Hours/ha	Estimated by Eng. Julio Lovera Fernández
	Wood extraction volume	48	m^3^/ha	Estimated by Eng. Julio Lovera Fernández

^a^1 US$ = 3.84 Soles

## Discussion

Proposing policies for the conservation of the forest system requires an understanding of the metabolic pattern of the socioeconomic system within its environment. In the case of Huayopata, the lack of alternative sources of energy to process tea may be playing against forest systems (Ortega Loaiza, 2013). The incentive for national tea production is at odds with forest conservation policies, on two fronts: (a) increasing/recovering the area used for tea crops and (b) increasing the requirement of firewood for the processing of tealeaves. In this sense, the annual production of fresh leaves projects a radical increase from 600 tons to 3840 tons in the next five years (local tea producer, personal communication). Therefore, it is equivalent to 768 tons of black tea/year (considering a ratio of 5 to 1 between fresh tealeaves and black tea), which entails an energy requirement of 2860 m^3^ of firewood/year ([768 t of black tea * 2.7 t of firewood/t of black tea]/[0.725 t of firewood/m^3^ firewood]. At this point, if we use the estimated timber volume per hectare as an example (see Table 2), it would then represent a deforestation of 60 hectares of secondary forests ([2860 m^3^]/[48 m^3^/ha]). Moreover, the rebound in national tea production may cause an increase in the number of migrant workers, who will require an additional local food supply. Evidently, this household sector growth will exert greater pressure on the agricultural and forest systems owing to an expansion of the agriculture frontier and the requirement of firewood for cooking purposes. This can appear to be paradoxical given that the Camisea natural gas field, which accounts for practically all the proven Peruvian reserves (Dammert Lira & Molinelli, 2006), is located in the Echarate district, less than 100 km from Huayopata. Moreover, in 2021, given the increase in fuel prices, it was reported that a 10-kg LPG container in the communities near to Huayopata costs between 80 and 100 Soles (21–26 US$) (La República, 2021), which almost doubles the selling price in Lima—which is a ca. 1000 km-drive from the deposit. This illustrates the uncomfortable knowledge pointed out by (Rayner, 2012) and studied in David Pimentel's works (Pimentel & Pimentel, 2008).

The MuSIASEM shows interactions between the socioeconomic system and the ecological system, since the abandonment of agricultural activities will give way to a recovery of the forests, including a transition from secondary forests to mature forests. Although this is desirable from an ecological standpoint, the underlying migration and population aging situation poses future risks of:

- Economic self-sufficiency. This subject generates a discussion on the funding sources to maintain/improve the quality of life of the people who remain in the district and, also, on how much and who will pay for them (Economic externality). A society that is economically dependent on government aid is subject to persistent economic vulnerabilities, due to changes in policies and external situations.
- Food self-sufficiency. Agriculture not only provides an economic income but also the necessary food that guarantees Huayopata's social stability. Reducing food production means that it will need to be produced elsewhere, since no one can survive without food; hence, the discussion turns to *how much*, *where*, and *who* produces these foods. In addition, the MuSIASEM visualizes that importing and exporting food entails the import and export of the inputs that are required for production. Therefore, the discussion must include what pressure and impact is exerted on these other ecosystems (biophysical externality).

Thus, the MuSIASEM enriches the debate by integrating topics that are generally ignored or underestimated and that cause new problems of a different nature over time. It is precisely these types of situations that we are faced with when it comes to sustainability, where there are no win–win recipes. However, making trade-offs transparent makes social stakeholders aware of who will and who will not benefit, avoiding future conflicts and unexpected events.

Another potential of the MuSIASEM is that its versatility to gradually adapt to different contexts and incorporate systems and elements of interest that cover different sectors facilitates the cross-sectoral policy-building process, thus addressing criticism regarding the bias of nexus approaches (Smajgl et al., 2016). For example, the Law that promotes tea production, which will have a notable impact on Huayopata's agricultural activity, is at odds with the guidelines of Peru's government to reduce deforestation. However, findings suggest that this conflicting situation has not yet emerged—at least not for the time being—not because of competition for land resources, but rather because of a lack of access to cheaper and cleaner energy sources. Consequently, strategies to recover the tea production activity must be followed by adequate energy policies. People who work in the tea industry have a legitimate right to work in order to maintain their well-being. Therefore, this type of model highlights the need for alliances between the public sector (which provides legal and economic instruments), the academia (which provides scientific and technological knowledge) and the industry (which provides experiential knowledge).

Additionally, the above conflicting situation suggests the importance of addressing the relational analysis against the issue of scale and level, given that it illustrates how policies adopted at the national-level impact on communities (zonal scale) and how the consequences that occur on this zonal scale (for example, an increase in deforestation) influence the objectives at a national scale. In this way, the MuSIASEM bridges the gaps that have been detected in the nexus analyses regarding vertical integration between the local and the national and vice versa (Endo et al., 2017).

A limitation of the MuSIASEM that is common to other approaches based on nexus analyses is the availability of information to cover all the necessary variables (Shannak et al., 2018). However, that which appears to be a weakness can also be interpreted as a strength, in the sense that the lack of information cannot serve as a justification for disregarding a topic. Quite the contrary, the identification and justification of the reason why this information is important should promote a joint effort to gather the missing information. In addition, the organization of information through matrices generated by the MuSIASEM allows us to align the missing values and inquire about events that are occurring but are not being mapped—for further details see (Giampietro & Bukkens, 2015). Also, this type of information can be coupled with geographic information systems (GISs) to territorialize the information, thereby identifying potential issues. For example, cross-referencing information on excessive fertilizer use with groundwater sources can help to identify potential contamination problems. Finally, in light of the fact that the MuSIASEM is a system capable of quantitatively operationalizing the metabolic pattern of the systems under study, it contributes to evolving from a "nexus thought" toward a "nexus" (Simpson & Jewitt, 2019).

## Conclusions

This paper presents a methodology based on the principles of David Pimentel and the MuSIASEM to simultaneously study the society–agriculture–forest system. Although agricultural activity has been identified as one of the main drivers of land use, causing deforestation and loss of biodiversity, it also serves a specific purpose, namely to produce food in order to ensure food security and, as a merchandise, to obtain funds to preserve farmers' welfare. Consequently, any change in the behavior of the five components included (local socioeconomic system, external socioeconomic system, the agricultural sector, and the primary and secondary forest) will affect the rest. In this sense, the proposed model identifies and explores these relationships to broaden the discussion aimed at protecting forests and moving toward a sustainable development. In addition, the model enables the characterization of the agricultural system based on extensive and intensive variables that can prove useful for resource management. However, one of the main limitations of the model is the amount of information required, which often does not exist or is not readily available. For this reason, the results of the Huayopata study should be interpreted with caution, given that the COVID-19 pandemic limited the scope of field work. Consequently, a more robust analysis is required. Despite this, the information obtained serves the purpose of proving the model's usefulness.

As regards the case study, results obtained suggest a decrease in Huayopata's population due to the effect of migration, which may be reducing the agricultural activity, leaving room for the growth of secondary forests. If this trend continues and a decision is made to protect secondary forests, it will take almost a century for the latter to recover some of the attributes of primary forests.

The dietary pattern has also an impact on productive systems and ecosystems. Therefore, any policy aimed at improving people's nutrition and/or improving food security—it must be noted that Huayopata is dependent on food from other regions of the country or from abroad, given that it is 29% self-sufficient in terms of plant-based food and 28% self-sufficient in terms of animal-based food—will also be a driver for the agricultural activity seeking to recover the spaces left to secondary forests or expand the agricultural frontier. Also, policies adopted to recover the main economic activity in the area, that is, tea production, endanger the forestry system. The reason is twofold: (1) the reactivation of this activity can be an immigration 'pull factor', and immigrants' basic needs, such as food, will need to be met, which in turn will drive the agricultural activity, and (2) the cauldrons used for tea processing work on firewood obtained from forests, therefore promoting this activity will exert greater pressure on this ecosystem.

## Supplementary Information

Below is the link to the electronic supplementary material.

Supplementary file1 (XLSX 41 kb)
